# Supplementary material for: Challenges in implementing the WHO-recommended package of care for advanced HIV disease in resource-constrained settings: A mixed-methods study
Source: PLoS One. 2026 Jan 20;21(1):e0341162. doi: 10.1371/journal.pone.0341162 (PMC12818689; doi:10.1371/journal.pone.0341162)
Supplement: S1 Table — (DOCX) [file pone.0341162.s001.docx]

**S. Table 2. Codebook on challenges in implementing the WHO-recommended package of care for AHD**

| **Name** | **Description** | **Files** | **References** |
| --- | --- | --- | --- |
| **Patient-related concerns as expressed by healthcare workers** |  | **6** | **10** |
| Clinical factors | Most AHD patients require clinical stabilization before ART initiation, causing delays. | 1 | 1 |
| Financial constraints | AHD patients with low socio-economic status cannot afford to buy medicines from retail drug outlets and many remain untreated. | 5 | 6 |
| Psychological unreadiness | Some patients adamantly reject the test results, delaying their enrollment in ART. | 2 | 3 |
| **Service delivery constraints** |  | **8** | **25** |
| High workload and inadequate human power | High workload from routine tasks and a large patient load in the ART clinic negatively impacts quality of care. | 4 | 6 |
| Inadequate service availability | Limited availability of services, such as baseline testing, hampers accurate diagnosis and effective management. | 4 | 9 |
| Ineffective referral and linkage | The referral system is poorly structured and inconsistent, hindering the timely provision of essential services. | 3 | 4 |
| Insufficient training | Insufficient professional training on AHD leads to misdiagnosis, improper treatment, and reduced quality of care. | 5 | 5 |
| Underutilization of services by spoke sites | Essential services available at the hub center are underutilized by spoke facilities. | 1 | 1 |
| **Structural and organizational obstacles** |  | **8** | **45** |
| APTS-related challenges | The restriction of within-facility drug exchange practices under the APTS system hinders access to OI medications. | 1 | 2 |
| Lack of specialized AHD clinics | AHD patients do not receive adequate care due to the absence of differentiated AHD services. | 2 | 3 |
| Non-comprehensive AHD registry | The limited scope of the AHD registry impedes detailed documentation of prognostic information. | 1 | 1 |
| Service interruptions due resource shortages | Resource shortages have caused interruptions in essential services, such as baseline CD4 testing and LF-LAM. | 8 | 15 |
| Supply chain constraints | Supply chain and logistical constraints limit the availability of essential medications and services. | 7 | 24 |
