# Supplementary material for: Challenges in implementing the WHO-recommended package of care for advanced HIV disease in resource-constrained settings: A mixed-methods study
Source: PLoS One. 2026 Jan 20;21(1):e0341162. doi: 10.1371/journal.pone.0341162 (PMC12818689; doi:10.1371/journal.pone.0341162)
Supplement: S2 Table — (DOCX) [file pone.0341162.s002.docx]

**S. Table 1. Components of the package of care for people with advanced HIV disease**

|  | **Intervention** | **CD4 cell count** | **Adults** | **Adolescents** | **Children**  **<10 years** |
| --- | --- | --- | --- | --- | --- |
| **Screening and diagnosis** | Screening tools for TB disease for adults and adolescents: WHO-recommended four-symptom screen, chest X-ray, C-reactive protein, WHO-recommended molecular rapid diagnostic test for TB, alone or in combination  Screening tools for TB disease among children: symptom screening for children living with HIV | Any | Yes | Yes | Yes  (Symptom screen only) |
|  | WHO-recommended molecular rapid diagnostics as the first test for pulmonary TB diagnosis  among those who screen positive for TB and investigations for extrapulmonary TB as applicable; chest X-ray may also be used to support investigations | Any | Yes | Yes | Yes |
|  | LF-LAM to assist TB diagnosis among people with symptoms and signs of TB | ≤200 cells/mm3 (inpatient)  ≤100 cells/mm3 (outpatient)  Or any CD4 count with symptoms or if seriously ill | Yes | Yes | Yes |
|  | Cryptococcal antigen screening | Recommended for  <100 cells/mm3 and  considered for 200 cells/mm3 | Yes | Yes | No |
| **Prophylaxis and pre-emptive treatment** | Co-trimoxazole prophylaxis | <350 cells/mm3 or  clinical stage 3 or 4  Any CD4 count in settings with  high prevalence of malaria or severe bacterial infections | Yes | Yes | Yes  For criteria, see Chapter 6 |
|  | TB preventive treatmenta | Any | Yes | Yes | Yes |
|  | Fluconazole pre-emptive therapy for cryptococcal antigen–positive people without evidence of meningitis | <100 cells/mm3 | Yes | Yes | Not applicable (screening not advised) |
| **ART**  **initiation** | Rapid ART initiationb | Any | Yes | Yes | Yes |
|  | Defer initiation if clinical symptoms suggest meningitis (TB or cryptococcal) | Any | Yes | Yes | Yes |
| **Adapted adherence support** | Tailored counselling to ensure optimal adherence to the advanced HIV disease package, including home visits if feasible | <200 cells/mm3 | Yes | Yes | Yes |

^a^TB preventive treatment should be provided in accordance with current WHO guidance.

^b^People receiving a positive WHO four-symptom screen should initiate ART while being evaluated for TB if clinical signs and symptoms of meningitis are absent.
