# Supplementary figures and images for: Challenges in implementing the WHO-recommended package of care for advanced HIV disease in resource-constrained settings: A mixed-methods study
Source: PLoS One. 2026 Jan 20;21(1):e0341162. doi: 10.1371/journal.pone.0341162 (PMC12818689; doi:10.1371/journal.pone.0341162)

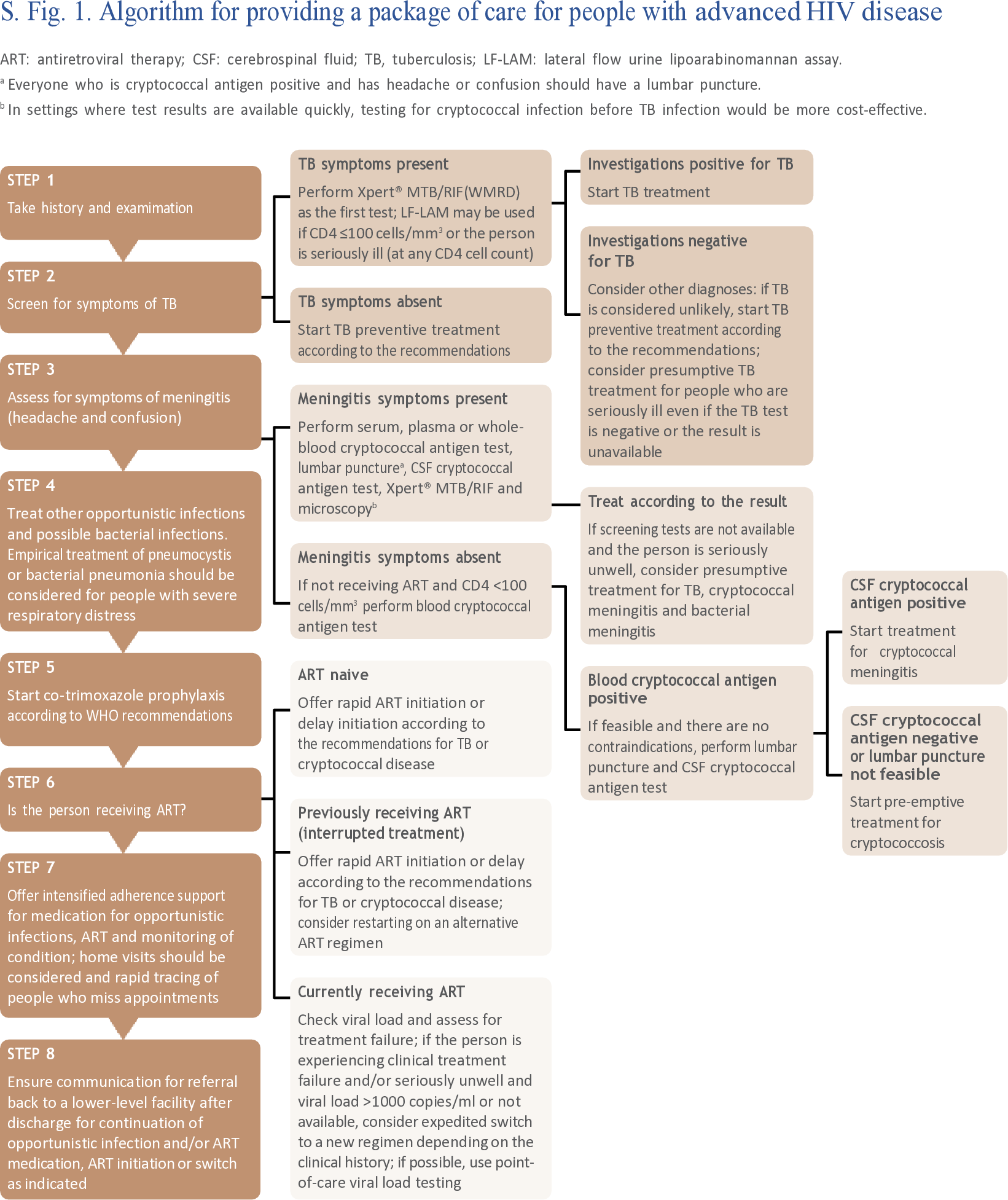

Supplement: S1 Fig — (TIF) [file pone.0341162.s003.tif]
