# Supplementary material for: Challenges in implementing the WHO-recommended package of care for advanced HIV disease in resource-constrained settings: A mixed-methods study
Source: PLoS One. 2026 Jan 20;21(1):e0341162. doi: 10.1371/journal.pone.0341162 (PMC12818689; doi:10.1371/journal.pone.0341162)
Supplement: S1 File — (DOCX) [file pone.0341162.s004.docx]

**Data collection tool**

**Title: Adherence to and Challenges in the Implementation of the WHO-Recommended Package of Care for Newly Diagnosed Advanced HIV Disease Patients in Resource-Constrained Settings: A Mixed-Methods Study**

**Part I: Data abstraction checklist**

Intervention-I: Screening and diagnosis

| S. No | Package of care |  | Performed | Result | Remark |
| --- | --- | --- | --- | --- | --- |
| 1. | TB Screening (*WHO-recommended four-symptom screen: current cough, fever, weight loss or night sweats*) |  | 1. Yes | 1. Positive 2. Negative | If the screening test is positive, perform diagnostic tests: |
|  |  |  | 1. No |  |  |
| 1.1 | - Sputum Xpert® MTB/RIF as the first test for TB Diagnosis |  | 1. Yes | 1. Positive 2. Negative |  |
|  |  |  | 1. No |  |  |
| 1.2 | - Urine LF-LAM test |  | 1. Yes | 1. positive 2. Negative | CD4 < 200; OPD  CD4 <100; IPD |
|  |  |  | 1. No |  |  |
| 2 | Cryptococcal Antigen screening |  | 1. Yes | 1. Positive 2. Negative |  |
|  |  |  | 1. No |  |  |

Intervention-II: Prophylaxis and pre-emptive treatment

| S. No | Package of care | Performed | Remark |
| --- | --- | --- | --- |
| 1. | Cotrimoxazole prophylaxis | 1. Yes |  |
|  |  | 1. No |  |
| 2. | TB Preventive treatment | 1. Yes |  |
|  |  | 1. No |  |
| 3. | Fluconazole pre-emptive therapy | 1. Yes | CD4 <100 |
|  |  | 1. No |  |

Intervention-III: ART initiation

| S. No. | Package of care | Performed | Remark |
| --- | --- | --- | --- |
| 1. | Components of the ART regimen: | 1. TD4/3TC/DTG 2. TD4/3TC/EFV-400 mg 3. TDF/3TC/EFV-600 mg 4. TDF/FTC/DTG 5. TDF/FTC/EFV-600 mg 6. AZT/3TC/EFV-600 mg 7. TDF/3TC/PI/r 8. TDF/3TC/RAL 9. TDF/3TC/DTG 10. TAF/3TC/DTG 11. ABC/3TC/DTG 12. Other |  |
| 2. | Number of days between diagnosis and initiation of ART | ____ (days) | Difference between ART initiation date and diagnosis date |

Intervention-IV: Adherence support

| S. No. | Package of care | Performed | Remark |
| --- | --- | --- | --- |
| 1 | Tailored counseling | 1. Given |  |
|  |  | 1. Not given |  |

**Part II: Interview Guide for in-depth interviews with KIs**

**Target Respondents**: Health professionals in ART Care Continuum.

**A. Background information**

1. Age:
2. Sex:
3. Profession:
4. Experience:
5. How long have you been involved in HIV care, particularly in managing AHD?

**B. Implementation challenges**

1. How are patients with AHD identified at your facility?
2. Do you have consistent access to CD4 testing?
3. Baseline CD4 count test is not conducted for significant proportion of PLHIV enrolling into ART. What are possible reasons for this gap?
4. Are there any challenges you have faced in diagnosing tuberculosis, especially related to Xpert® and LF-LAM?
5. Are there any challenges related to the diagnosis and management of OIs?
6. Is baseline CrAg testing performed for AHD patients? If not, what are the possible reasons for not conducting the CrAg test?
7. What are the possible (clinical, logistical, personal, or health system-related) reasons why some AHD patients did not start ART on the day of diagnosis or within seven days?
8. What are the main resource-related (supply chain) barriers to implementing the AHD care package, such as limited availability of equipment, diagnostic kits, or medications?
9. Can you tell me if there are any issues related to staff shortages or lack of training?
10. Have you or your colleague recently received training related to AHD? In what areas do you need more training or professional support?
11. Do Zonal or Regional health bureaus or partner organizations provide professional support and monitoring? If so, how?
12. What needs to be changed (improved) to provide better AHD treatment? If you have any general comments, please let us know.

**Thank you**
