# Supplementary material for: Challenges in implementing the WHO-recommended package of care for advanced HIV disease in resource-constrained settings: A mixed-methods study
Source: PLoS One. 2026 Jan 20;21(1):e0341162. doi: 10.1371/journal.pone.0341162 (PMC12818689; doi:10.1371/journal.pone.0341162)
Supplement: Attachment — Submitted filename: Response to Reviewers.docx [file pone.0341162.s006.docx]

**Rebuttal letter**

**Dr. Felix Bongomin, MB ChB, MSc, MMed, FECMM**
Academic Editor
PLOS One

Dear Dr. Felix Bongomin,

We sincerely thank you for your time and professional efforts in handling our manuscript. In response to the reviewers’ comments and in accordance with the journal’s guidelines, we have carefully revised the manuscript. The revised version is submitted along with this rebuttal letter. All modifications in the manuscript have been clearly highlighted in dark red for your convenience. In accordance with the journal office’s recommendation, the newly cited references are provided at the end of this letter for your review.

For clarity and conciseness, we have amended the title to: “Challenges in implementing the WHO-recommended package of care for advanced HIV disease in resource-constrained settings: A mixed-methods study”.

Below, we respond in detail to each comment, and we believe that the revisions incorporated effectively address the concerns and recommendations provided.

**Journal Requirements**:

When submitting your revision, we need you to address these additional requirements.

1. Please ensure that your manuscript meets PLOS ONE's style requirements, including those for file naming.

**Response**: We thank you for the opportunity to revise our manuscript. We have carefully revised the manuscript in response to the comments and suggestions from the academic editor and reviewers. The revised version now fully complies with PLOS ONE’s style requirements.

2. You have indicated that data is available from [[mohammedf@du.edu.et](mailto:mohammedf@du.edu.et)].  Please can we ask you to provide us with a general contact email address for the data requests, so readers can request access in perpetuity. If a general email is not available please provide a link to a website where readers can obtain access to data.

**Response**: We have updated the “Data availability statement” by replacing [mohammedf@du.edu.et](mailto:mohammedf@du.edu.et) with the general contact email address of the Institutional Review Board of the College of Medical and Health Sciences at Dilla University (IRB_CHSM DU): [duirbsubmission@gmail.com](mailto:duirbsubmission@gmail.com).

3. If the reviewer’s comments include a recommendation to cite specific previously published works, please review and evaluate these publications to determine whether they are relevant and should be cited. There is no requirement to cite these works unless the editor has indicated otherwise.

**Response**: We received a recommendation to cite the published version of an article that was previously included as a preprint (Ref. #32) in our initial submission. We have updated the reference accordingly.

4. Please review your reference list to ensure that it is complete and correct. If you have cited papers that have been retracted, please include the rationale for doing so in the manuscript text, or remove these references and replace them with relevant current references. Any changes to the reference list should be mentioned in the rebuttal letter that accompanies your revised manuscript. If you need to cite a retracted article, indicate the article’s retracted status in the References list and also include a citation and full reference for the retraction notice.

**Response**: We would like to clarify that none of the papers cited in our manuscript have been retracted. In addition, we have included relevant publications that further support our findings. A complete list of these newly added references is provided in the rebuttal letter below under ‘References that have been added to the revised manuscript’–Page^6^.

**Reviewer #1**:

**OVERALL COMMENT**
This is a very useful study describing implementation gaps in the public health approach to advanced HIV disease care in Ethiopia. I have suggested a number of minor comments that would help in tightening up the paper.

**Response**: Dear esteemed reviewer, we are profoundly grateful for the invaluable insights and detailed feedback you have so generously provided to enhance our manuscript.

One major suggestion would be for the investigators to be able to provide information on the vital status of the 145 study participants. The study was concluded over a year ago, so if possible it would be important to know if any participants had died. It would further be of interest to know timing (weeks since study end) and cause of death. But I appreciate not all of this information may be readily available.
A comment could also be made on what is generally known about HIV-associated mortality in the region/country.

**Response**: We appreciate your suggestion regarding the assessment of participants’ vital status. However, it was not feasible to track the vital status of participants because this was a cross-sectional study. Nevertheless, investigating treatment outcomes among individuals enrolled in antiretroviral therapy with advanced HIV disease remains an important focus of our future research.

Regarding HIV-associated mortality, data from the UNAIDS 2025 report have been added: *Globally, an estimated 630,000 HIV-related deaths occurred in 2024, of which approximately 60.3% were in the African Region*.

**SPECIFIC COMMENTS**
In 2023, an estimated 39.9 million people…
- These data should be updated (2024 data are available)

**Response**: Thank you for this observation. We have revised the manuscript to incorporate updated data from the UNAIDS 2025 report. (Lines: 55–58)

*At the end of 2024, approximately 40.8 million people worldwide were living with HIV, including 1.3 million new infections, of whom 31.6 million were receiving antiretroviral therapy (ART). Globally, an estimated 630,000 HIV-related deaths occurred in 2024, of which approximately 60.3% were in the African Region.*

“many individuals in SSA still cannot access treatment until the later stages of their illness, often presenting with advanced HIV disease”
- Data suggest that in many contexts the majority of people with AHD are not accessing treatment late, but rather are disengaging from care after having started ART. If possible, it would be helpful to describe the proportion who were newly diagnosed vs ART experienced.

**Response**: Dear Reviewer, a recent study ^[1]^ by de Waal et al., which examined global trends in CD4 count measurement and distribution at first antiretroviral treatment initiation, reported a concerningly high prevalence of AHD. It has reported the prevalence of AHD among individuals initiating ART with a baseline CD4 count ranged from 32% to 40% across four African regions, excluding South Africa.

*We have reported the proportion of AHD among newly diagnosed individuals initiating ART. (Lines: 66–68)*

“International funding has been crucial in reducing new HIV infections and related deaths in SSA”
- This section would better go in the Discussion

**Response**: We appreciate your suggestion that the above-mentioned section would be more appropriately placed in the Discussion. However, we believe that including it in the Introduction more effectively highlights the efforts undertaken thus far to end the AIDS epidemic. In response to your concern, we have also incorporated this point into the Discussion in relation to our findings, as follows: “*Suboptimal implementation of established recommendations, compounded by recent funding cuts, could reverse global efforts to end the AIDS epidemic as a public health threat by 2030.*” (Lines: 401–402)

“two-thirds (66.7%) had a CD4 count between 100 and 199. Of the 145 AHD cases, only 69 (47.6%) had a recorded baseline CD4 count”
- It needs to be clear that the 66.7% is a fraction of the 69 individuals who got a CD4.
It should also be stated how the remaining individuals were diagnosed with AHD. (In the discussion it is stated that “This implies that more than half of the cases were identified using WHO clinical staging”. Is this “implied”, or was it recorded by the investigators?)

**Response**: Dear Reviewer, thank you for raising your concern regarding clarification of the proportion of CD4 count and WHO clinical staging. In response to your query, all individuals diagnosed with AHD had documented WHO clinical staging, whereas only 69 individuals had a recorded baseline CD4 count. For those without a baseline CD4 count (n = 76), WHO clinical staging was used for classification. We have now incorporated the frequency and corresponding percentage of the variable “*WHO clinical staging*” in Table 1. Furthermore, we rephrased the sentence “two-thirds (66.7%) had a CD4 count between 100 and 199. Of the 145 AHD cases, only 69 (47.6%) had a recorded baseline CD4 count” to: “*Of the 145 AHD cases, only 69 (47.6%) had a recorded baseline CD4 count. Among these 69 cases, two-thirds (n = 46, 66.7%) had a CD4 count between 100 and 199 cells/mm³*” (Lines: 184–186)

Ref 32 is a pre-print and is now published: <https://pubmed.ncbi.nlm.nih.gov/41002116/>

**Response**: Thank you for bringing this to our attention. The published version has now been cited in place of the preprint ^[2]^.

“A key concern emerging from the qualitative findings is the limited availability of medications for the management of OIs.
- It is worth stressing that cryptococcus is particularly overlooked - none were tested for CrAG, only 33 received fluconazole primary prophylaxis.

**Response**: Thank you for pointing this out. We agree with your observation and have revised the manuscript accordingly. In revised manuscript we have emphasized the implications of gaps in CrAg test and fluconazole primary prophylaxis. (Lines: 458–465)

“Median (IQR) duration from diagnosis to ART initiation was 15 days (14–18).”
- Under “Clinical and public health implications” this could be stressed as a simple area for improvement. It is stated that for some patients enrollment into ART is postponed until they come to terms with the diagnosis and are psychologically prepared to begin treatment. The presented data suggests this is the case for all patients.
- It is noted that “Prior to initiating ART, healthcare providers are advised to conduct a comprehensive discussion with patients…”. It could be emphasized that much of this counselling can be done in parallel to ART initiation, rather than sequentially. (The key issue is to rule out the risk of IRIS (screen for TB, assess for symptoms of meningitis)

**Response**: We appreciate your professional suggestion and have incorporated a sentence emphasizing the immediate initiation of ART, accompanied by counseling. (Lines: 522–524)

Patient-related concerns are cited, but only providers were interviewed. This is noted as a limitation, but it could also be made clearer in the text (e.g. “Theme 3: Patient-related concerns as expressed by healthcare workers”

**Response**: We appreciate your expertise and have revised “Theme 3” for clarifications in response to your recommendations. Accordingly, we have updated the manuscript and the accompanying supporting information file (the codebook).

Dear Reviewer, we are grateful for your expertise and the professional effort you have invested in enhancing our manuscript. We hope that the revisions we have made meet your expectations and address the concerns you raised.

**References that have been added to the revised manuscript**

1. de Waal et al.^[3]^
2. Stover et al.^[4]^ (*pre-print*)
3. Endalamaw et al.^[5]^
4. Lujintanon et al.^[6]^
5. Maphosa et al.^[7]^
6. Chachu and Maboe ^[8]^
7. Tukamuhebwa and Gemmell ^[9]^
8. Msowoya et al.^[10]^
9. Chachu and Maboe ^[11]^
10. Ahmed et al.^[12]^
11. Govere et al.^[13]^
12. Chueh et al.^[14]^

1. <https://doi.org/10.1093/cid/ciae548>
2. <https://doi.org/10.1111/hiv.70122>
3. <https://doi.org/10.1093/cid/ciae548>
4. <https://doi.org/10.2139/ssrn.5199076>
5. <https://doi.org/10.1186/s12889-024-19016-5>
6. <https://doi.org/10.1002/jia2.26263>
7. <https://doi.org/10.1186/s12889-025-24157-2>
8. <https://doi.org/10.1111/hiv.70022>
9. <https://doi.org/10.1186/s12879-025-10682-3>
10. <https://doi.org/10.1186/s12981-025-00725-9>
11. <https://doi.org/10.1371/journal.pone.0309579>
12. <https://doi.org/10.1186/s12889-020-09887-9>
13. <https://doi.org/10.1186/s12913-023-09801-0>
14. <https://doi.org/10.1016/j.jiph.2025.102677>
